# Levelling the playing field in assessment: an analysis of attainment gaps for widening participation, black and minority ethnic mathematics undergraduates before and after the COVID-19 lockdown

**DOI:** 10.1093/teamat/hrab024

**Published:** 2021-10-29

**Authors:** L M Shaw, M R Tranter

**Affiliations:** Nottingham Trent University, 50 Shakespeare St, Nottingham NG1 4FQ, UK

## Abstract

The 2019/20 Level 4 mathematics cohort at the Nottingham Trent University sat a full set of mid-year assessments in January 2020 under completely normal circumstances. However, the Covid-19 lockdown meant that their end of year assessments, along with all of their teaching and learning from March 2020 onwards, moved fully online. This has given us a unique opportunity to understand how the same cohort perform in contrasting situations. In this study we consider the issue of attainment gaps and find that the attainment gap closed in this cohort for black and minority ethnic students but that students from a lower socio-economic background may have been put at a disadvantage by the move to online teaching, learning and assessment. We use a linear mixed effect models approach to present statistical evidence to support these two claims as well as investigating the specific aspects of the move online, which may have caused these results.

## 1 INTRODUCTION

Attainment gaps (also known as awarding gaps) are a major issue across the UK higher education sector and mathematics is no exception to the problem (Hunter *et al.*, 2018). At present, students from non-white or lower socio-economic backgrounds, students with disability or those who enter higher education with business and technology education council qualifications generally have lower attainment than their peers. There has been investigation into the root causes and potential solutions for each of these attainment gaps Cliffe *et al.* (2020); Gallimore & Stewart (2014); Panesar (2017). In mathematics, this has largely come down to making use of support centres or drop-in sessions and targeting students who are deemed at high risk of not progressing in their degree (Grove *et al.*, 2020). Recent studies have shown that the attainment gap for children from lower socio-economic backgrounds has widened as they struggled during the move to home learning (Andrew *et al.*, 2020; Bonal & González, 2020), but little seems to have been published regarding how attainment gaps in higher education have been affected during the Covid-19 pandemic.

In 2019, the National Union of Students and Universities UK released a report suggesting that ‘Universities need to take a more scientific approach to tackling the attainment gap, by gathering and scrutinizing data in a far more comprehensive way than they may currently be doing, in order to inform discussions between university leaders, academics, practitioners and students’ (NUS and UUK, 2019). This report was aimed specifically at addressing the attainment gap for black, Asian and minority ethnic students. In this paper, we aim to use a data-oriented approach to understand a range of attainment gaps that exist in mathematics at the Nottingham Trent University and to investigate whether there are any lessons to be learned from the move to online teaching, learning and assessment as a result of lockdown. It is hoped that these lessons may be applicable to mathematics departments at other universities, and even outside of mathematics.

We look to answer the following research questions:

- Was there a significant change in attainment gaps for black and minority ethnic (BME) or other widening participation students as a result of the move online in March 2020?
- Where attainment gaps did close/widen, were these consistent across all modules or did modules with certain assessment types see different results?
- Can lessons be learned from this analysis to help close attainment gaps in future?

We begin by presenting a review of literature surrounding attainment gaps in the wider higher education sector in the UK, looking at strategies to both analyse and address the issue. We then present the research methodology for this paper, explaining the nature of the available data, before describing and justifying transformations to the data and the linear mixed effect model approach used in this analysis. Finally, we present the results of this analysis and discuss the findings, in order to address the three research questions above.

## 2 LITERATURE REVIEW

Attainment gaps, especially those for BME students, have been a key issue for some time in UK higher education. It has long since been established that such gaps exist (Connor *et al.*, 1996), but methods for analysing these gaps and establishing their extent and significance have largely been confined to the visual analysis of tables and charts, as well as making use of relatively basic statistical tools such odds ratios (Richardson, 2018). However, some more sophisticated techniques have been employed when looking at this issue. Broecke & Nicholls (2007) report for the UK government Department for Education and Skills used an ordered logistic regression approach to find that ethnicity, gender, disability and prior qualifications all had a significant impact on student attainment at university (Broecke & Nicholls, 2007).

In 2020, a study of nearly 150 000 distance learning students at the Open University from Nguyen et al. found that attainment gaps still exist for BME students in this setting (Nguyen *et al.*, 2020). Importantly, the authors were able to control for other factors (such as engagement) by using a mixed linear model method and still found that the BME attainment gap persists. The ability to control for other factors is a key reason why we adopt a similar approach in our own analysis.

While the literature is clear that attainment gaps exist, pinpointing the root cause have proven to be more difficult. In a review paper specifically addressing the BME gap, Richardson notes that the gaps differ across institutions and courses, suggesting that there are teaching and assessment practices that may influence attainment gaps, but also noting that pinpointing the specific practices that lead to changes in attainment gaps is an ongoing challenge (Richardson, 2018). Cotton et al. suggest that BME and male students may be more likely to use surface level learning approaches and are potentially more likely to overestimate their current attainment levels and therefore, also their chances of future success. However, they also point out that these are simply issues that may warrant further explanation Cotton *et al.* (2016). By comparing attainment gaps in the same cohort across two different teaching, learning and assessment styles, it is hoped that this paper may go some way to providing further insight into understanding which specific practices may contribute to closing or widening attainment gaps.

## 3 METHODS

### 3.1 Available data

We have data from 114 Level 4 students at the Nottingham Trent University in 2019/20 who were taking one of the mathematics cluster of courses. Among these students, 29 of them identified as being from a black, Asian or minority ethnic background and 24 met the criteria to be considered as widening participation based on their socio-economic background, decided by indicators such as household income and participation of local area rates in higher education. We use the abbreviation BME to denote students from a black, Asian or minority ethnic background and WP to denote the students who are considered to be widening participation due to their socio-economic background. There were 11 students who were categorized as both BME and WP. These data are summarized in Table 1.

**Table 1:** Categorization of students in the study

	BME	non-BME	**Total**
WP	$11$	$13$	$24$
non-WP	$18$	$72$	$90$
**Total**	$29$	$85$	$114$

For each student, we have the result of every summative assessment from each of the six modules taken by the BSc Mathematics cohort. These results are all given as a percentage. For each assessment, the following information was also recorded:

- The module that the assessment came from. Modules are labelled **A**, **B**, **C**, **D**, **E** and **F**.
- The timing of the assessment. **Pre** or **Post** lockdown. Each module had one assessment at each time point.
- The nature of the assessment. Coursework (**CWK**) assessments did not change as a result of the move to online learning. Examinations (**EXM**) pre-lockdown were closed-book but post-lockdown these were altered to become 24-hour take home, open-book assessments (**24H**).

The assessment types for each module are given in Table 2.

**Table 2:** Pre- and post-lockdown assessment types and student numbers by module

Module	Pre	Post	Took module	Sat both assessments
A	CWK	CWK	$107$	$94$
B	CWK	24H	$53$	$49$
C	CWK	24H	$114$	$106$
D	EXM	24H	$84$	$75$
E	EXM	24H	$114$	$106$
F	EXM	24H	$60$	$56$

### 3.2 Considerations of the data

There are several aspects of the data to consider before performing our analysis. Firstly, not all students took all of the modules, as seen in Table 2. This came down to their choice of course, of which there are several, but which can be split into three reasonably clear groups.

- Maths students who took all six modules (53).
- Financial Maths and Data Science students who took modules A, C, D and E (31).
- Joint honours students, who only took three of the six modules. All students took modules C and E, the third module depended on the other half of their degree (30).

Another issue is that not every student sat all of their assessments, meaning that there are some zeros in the data. In most cases, the student gave valid reasons for their absence and were able to sit the paper again, uncapped in Summer 2020. However, we are specifically interested in comparing the performances of students in the mid-year and end of year assessment periods. Any results from a module in which the given student had not sat both assessments were removed from the data. Again, this information is provided in Table 2.

**Fig. 1.**
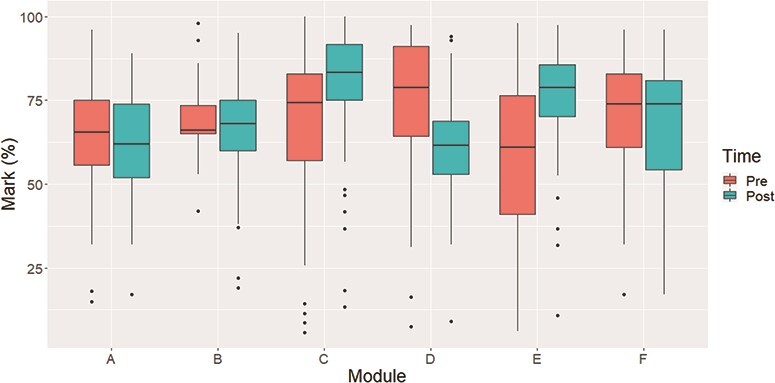
Distribution of marks for both the pre- and post-lockdown assessments in each of the six modules.

The distribution of marks in each of the twelve assessments is given in Fig. 1. We observe that there is a clear difference in the difficulty of some assessments but that this does not appear to be determined by the time of assessment in general. For example, in modules C and D, the post-lockdown assessment has higher marks but the opposite is true for module D.

**Fig. 2.**
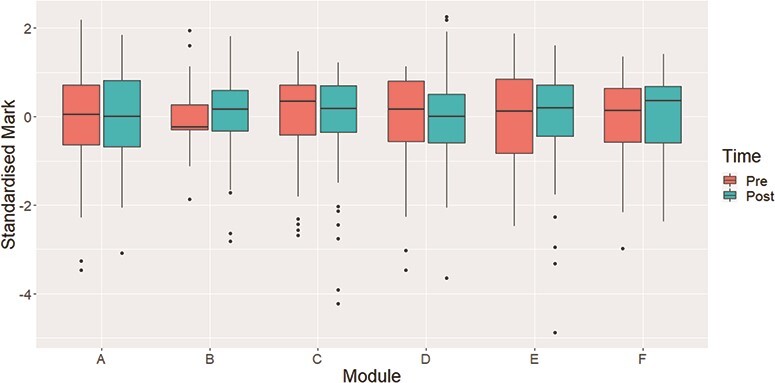
Distribution of standardized marks for both the pre- and post-lockdown assessments in each of the six modules.

The decision was taken to standardize the raw marks so that each assessment had a mean of $0$ and a variance of $1$. The justification for this is that our analysis is targeted at looking for differences in the performances of BME and WP students and assessments with higher variance in their results are more likely to influence this analysis. By standardizing the results, the scores from each assessment will be on a comparable scale and ensures that they each have a more equal contribution to the analysis. This is standard practice in statistics (James *et al.*, 2013) and the standardized distribution of marks for each assessment can be seen in Fig. 2. We observe that there are still some differences in the distributions, especially where lower marks are concerned but all twelve are roughly on the same scale.

### 3.3 Linear mixed model analysis

The standardized marks are the response variable for our data. Each mark is associated with a particular student, along with their BME and WP categorization, and a particular assessment, along with its time and module. While we have used standardization to ensure that the marks from each assessment behave in a similar manner, we cannot analyse this response using standard measures, such as a t-tests, due to repeated measures from the same student, breaking the assumption of independence of data points. To get around this, we use mixed linear effects models, with student identity included as a random effect to be controlled for. The use of mixed linear models here allowed us to make use of all of our data, while ensuring that we account for having several data points from each student, which are not independent.

A total of six linear mixed effect models were fitted to the data, all of which aimed to tackle the first two research questions outlined in Section 1. Each model contained student identity as a random effect as well as two fixed effects. The first fixed effect was whether a student fell into a certain category (BME or WP). If this effect was deemed to be significant, this would provide evidence for an attainment gap between students that did and did not fall into a particular group. The second effect included in each model was one of three different aspects of the assessment. These aspects were the timing (pre- or post-lockdown), the assessment type ($CWK, EXM, 24H$) or the assessment itself ($A_{Pre}, A_{Post}, \dots , F_{Post}$). Due to the standardization of marks across all assessments, none of these second effects should have been significant on their own, however, including an interaction term between the two fixed effects allowed us to determine whether there was evidence for the timing, type of assessment or a specific assessment having a significant impact in widening or closing attainment gaps for BME or WP students.

The models were all fit using the *lme4* package in R (R Core Team, 2021; Bates *et al.*, 2015), with p-values to test for the significance of coefficient estimators provided by the *lmerTest* package (Kuznetsova *et al.*, 2017). Once fit, residuals for each model were tested for normality using the Shapiro–Wilk test to ensure that this assumption of a linear mixed model was not violated. In all six cases, the p-value for this test came out $<10^{-7}$. As a result the decision was taken to transform the raw marks by squaring their value before applying the standardization described in Section 3.2. Refitting the mixed effect models to the standardized and transformed marks produced approximately normally distributed residuals in all cases. Figures were created using *ggplot2* (Wickham, 2009).

## 4 RESULTS

Table 3 shows the output from fitting the two linear mixed models described in Section 3.3 with time of assessment as the second fixed effect to the standardized squared marks. Table 3 shows the variable, the estimated effect of that variable on the standardized mark in any given assessment and a p-value for the significance of the effect.

**Table 3:** Variable, effect size and p-value for linear mixed effect models with time of assessment as one fixed effect and either BME (left) or WP (right) categorization as the other fixed effect

Variable	Effect size	p-value	Variable	Effect size	p-value
BME	$-0.3648$	$0.0333$	WP	$0.0728$	$0.6915$
BME & Post	$0.3225$	$0.0056$	WP & Post	$-0.2883$	$0.0219$

First, note that the intercept and time variables have not been included in Table 3. As expected, these effects were very close to zero and not significant. Since the raw marks were squared and standardized, the magnitude of the effect size has little meaning but a significantly negative effect suggests that marks in a given assessment were lower when the variable category was met, and a significantly positive effect suggests higher assessment marks. For these models we see a significant BME attainment gap in general, but that that this attainment gap was virtually wiped out in post-lockdown assessments. The p-value of $0.0056$ for this interaction effect provides significant evidence that lockdown helped to close the BME attainment gap. On the WP side, we observe that there is no evidence for a general attainment gap for WP students among this cohort of students but there is evidence that a gap did emerge as a result of lockdown. Diagnostics were run on both models to ensure that their residuals met normality assumptions and the Shapiro-Wilk test gave p-values of $0.41$ and $0.23$ for the BME and WP models, respectively, providing no cause for concern that normality assumptions were violated.

The next pair of models fit used assessment type (as given in Table 2) as the second effect and the results from these are given in Table 4. Again, all intercept and assessment effects were close to zero and not significant so have not been included. The only significant effect in the BME model is the interaction term with the closed-book examination style assessment. This effect was negative suggesting that closed-book exams could be the cause of attainment gaps among BME students. For WP students, both the closed-book and 24-hour style examinations show evidence of potentially widening their attainment gap. The effect size of $-0.4541$ for WP students taking 24 hour closed-book exams is the largest among all four models considered in Tables 3 and 4 and has the smallest p-value. Both models from Table 4 also passed diagnostic testing, with Shapiro–Wilk p-values of $0.29$ and $0.28$ for the BME and WP models respectively.

**Table 4:** Output from fitting linear mixed effect model with assessment type and either BME (left) or WP (right) categorization as the fixed effects

Variable	Effect size	p-value	Variable	Effect size	p-value
BME	$-0.1389$	$0.4360$	WP	$0.2008$	$0.2937$
BME & EXM	$-0.3874$	$0.0114$	WP & EXM	$-0.3608$	$0.0261$
BME & 24H	$0.08764$	$0.5096$	WP & 24H	$-0.4541$	$0.0015$

The final pair of models considered used the assessment itself as an effect along with either BME or WP categorization. Due to having $12$ assessments, each model fitted $24$ effect terms (with interactions included). The smallest p-value for an effect across both the BME and WP models was $0.0419$, which cannot be considered significant given the level of multiple testing required for these models. As such, we cannot conclude that any particular assessment saw a significant widening or narrowing of attainment gaps for BME or WP students.

## 5 DISCUSSION

We have presented a linear mixed effect models approach to try to understand the effect that the move to online teaching, learning and assessment had on BME and WP student attainment, using pre and post-lockdown summative assessment results from the 2019/20 Level cohort of mathematics students at the Nottingham Trent University. In Section 1, we presented three research questions for this paper and we now discuss whether our analysis has shed any light on the answers to these questions.

The first question asked whether the move to online learning in March 2020 had an impact on attainment gaps for BME and/or WP students? In Table 3 we saw that BME students experienced a significant attainment gap in pre-lockdown assessments but were able to almost completely close this gap post-lockdown. On the other hand, WP students did not suffer any attainment gap in pre-lockdown assessments but that a significant gap did open up post-lockdown. Our second question asked whether and gaps that changed after lockdown could be attributed to certain modules, assessments or assessment types. Assessments were split into three categories: coursework, closed-book examinations, 24-hour take home examinations and coursework. Of these evidence was found that closed-book examinations, used pre-lockdown, widened both the BME and WP attainment gap and that 24-hour take home examinations widened the WP attainment gap.

Within these results, the positive effect of the move away from closed-book examinations is easier to explain. While there do not appear to be any studies explicitly looking at the relationship between the BME attainment gap and closed-book examinations, it has been found that a greater proportion of students feel more suited to open book assessments Williams & Wong (2009) and, in a mathematical context, open book examinations are widely regarded as reducing student anxiety Block (2012). It is possible that BME and WP students benefit the most from both of these aspects of open book examinations.

The opening up of an attainment gap for WP students is more difficult to explain since it is unclear whether this was caused by the move of teaching online or by the 24-hour examination assessment style, which made up for five of the six post-lockdown assessments. One possible cause of this may be the sheer pace of the move to fully online learning, which happened in the space of one week in March 2020. WP students are less likely to be well connected digitally (Cannell & Macintyre, 2017). Specifically, this could refer to poor internet connections to access online classes or not having access to a laptop at all. While the Nottingham Trent University did provide a laptop loan scheme, it did take some time for all students to be access this, potentially causing some WP students to fall behind in their studies.

In the future, we will look to follow the fortunes of the cohort used in this study as they progress in their degree. First to Level 5 in 2020/21, which was almost fully taught and assessed online, and ultimately to Level 6 year in 2021/22, in which they should be returning to a more campus-based style of teaching, learning and assessment. It will be interesting to see how the attainment gaps change for this cohort under each of these conditions. For example, does the WP attainment gap for online learning hold when students are able to access laptops through the laptop loan scheme in preparation for the academic year, rather than in the middle of term?

In terms of our final research question, the lessons to be learned from this analysis appear to be to closely monitor the effect on closed-book examinations on attainment gaps and to potentially consider moving some of these examinations to open-book style assessments on a more permanent basis if further evidence is found that this may close the attainment gap. Universities also need to ensure that if elements of online teaching, learning and assessment are preserved in future, that all students have the means to readily access the online material.

Another area for further research could be to consider how other under-represented groups, such as disabled students or those who enter the degree with non-traditional qualifications performed. We were unable to look at these groups for this study since these groups contained $8$ and $7$ students respectively in the cohort that we used but conducting a study with a wider data set would allow us to develop an understanding of how lockdown affected these students. This could involve extending the scope of the study to a wider range of degree courses, or collating data on mathematics students from several institutions.

## Notes

**Laurence Shaw** has been a lecturer in mathematics and statistics at the Nottingham Trent University since 2017. His pedagogical research interests are the development of active learning environments, widening participation in HE, the relationship between HE mathematics students and their employability and understanding the effect of mathematics anxiety on HE students. His other research interests include epidemic modelling and antimicrobial resistance.

## Notes

**Matt Tranter** has been a lecturer in Applied Mathematics at the Nottingham Trent University since 2019. His pedagogical research interests are the development of active learning environments and the use of alternative assessments, such as Learning for Mastery and open book assessments, to help address attainment gaps. His other research interests include the study of nonlinear waves and scattering problems in layered waveguides.
